# Praise and Blame

**Published:** 1916-03-25

**Authors:** 


### PRAISE AND BLAME.

It will be generally agreed that opportunity and authority imply responsibility, or, to quote an ancient word, "unto whomsoever much is given, of him much shall be required." The precept applies in the medical world as it does elsewhere. Considered generally, the profession, though not unconscious of failures and errors, need not shrink from this trial, more especially in these modern days when the testimonies offered to the devotion and abilities of its members engaged in national service are both numerous and authoritative. It would be churlish to minimise the sincerity and value of these testimonies, or to receive them otherwise than with gratitude and goodwill. At the same time, it is well to remember that the profession has its own standard both of duty and of achievement, and by this and this alone it will elect to be judged. The reasons for this claim are, first, that only from within its own ranks is a competent judgment possible; and, secondly, that the standard so determined is not a fixed and rigid quantity but is ever rising with the demands and knowledge of the hour. Again, a final verdict on any given issue demands an appreciation of all the circumstances, and this, apart from technical knowledge and the chastenings of experience, is simply impossible. Hence, while it is pleasant to read that both soldiers in high command and representatives of the political hierachy recognise the good results which have been secured by the medical and surgical services rendered to the armies in the field, there will, we fancy, be little disposition in the profession to accept these voices as final, or to conclude from them that the best possible has been attained. Sooner or later all the medical questions which have been raised by the war must come for review before a competent professional tribunal, and if from such a source the verdict is one of undiluted eulogy the decision will be little deserving of confidence. Nor will it indicate much degree of insight, for quite certainly mistakes have been made, and if out of these we may not learn for the future we are indeed without help. The chance of such an issue may be placed with confidence among the impossibilities.

The chorus of congratulation is not without its dangers, though we are sure these are but for the moment. That it has recently been interrupted by a somewhat vigorous criticism by a member of Parliament will certainly not excite either ill-will or resentment. Indeed, we may go further and say that the sole concern of the profession is to make its work increasingly efficient in the special circumstances created by the war, and that any suggestion which will help to realise this ambition is to be welcomed. The individual points made by the critic would appear to have been disposed of to the full satisfaction of the official mind, but the profession itself will make its own independent and impartial inquiries. It will shape its conclusions by a no less standard than the best and most informed professional judgment, and, in so far as errors are detected, these will be opportunities for a move to higher levels. The demand of the moment is not to heed too much either praise or blame, but to discover the teachings of experience and to utilise these for ends not unworthy of the highest traditions.

